# Heat Shock Protein-Derived T-Cell Epitopes Contribute to Autoimmune Inflammation in Pediatric Crohn's Disease

**DOI:** 10.1371/journal.pone.0007714

**Published:** 2009-11-02

**Authors:** Gisella L. Puga Yung, Meredith Fidler, Erika Albani, Naomi Spermon, Gijs Teklenburg, Robert Newbury, Nicole Schechter, Theo van den Broek, Berent Prakken, Rosario Billetta, Ranjan Dohil, Salvatore Albani

**Affiliations:** 1 Arizona Arthritis Center, University of Arizona, Tucson, Arizona, United States of America; 2 Department of Medicine/Pediatrics, University of California San Diego, La Jolla, California, United States of America; 3 Department of Immunology, Institute of Biomedical Sciences (ICBM), School of Medicine University of Chile, Santiago, Chile; 4 EUREKA Institute for Translational Medicine, Siracusa, Italy; Julius-Maximilians-Universität Würzburg, Germany

## Abstract

Pediatric Crohn's disease is a chronic auto inflammatory bowel disorder affecting children under the age of 17 years. A putative etiopathogenesis of Crohn's disease (CD) is associated with disregulation of immune response to antigens commonly present in the gut microenvironment. Heat shock proteins (HSP) have been identified as ubiquitous antigens with the ability to modulate inflammatory responses associated with several autoimmune diseases. The present study tested the contribution of immune responses to HSP in the amplification of autoimmune inflammation in chronically inflamed mucosa of pediatric CD patients. Colonic biopsies obtained from normal and CD mucosa were stimulated with pairs of Pan HLA-DR binder HSP60-derived peptides (human/bacterial homologues). The modulation of RNA and protein levels of induced proinflammatory cytokines were measured. We identified two epitopes capable of sustaining proinflammatory responses, specifically TNF〈 and IFN© induction, in the inflamed intestinal mucosa in CD patients. The responses correlated positively with clinical and histological measurements of disease activity, thus suggesting a contribution of immune responses to HSP in pediatric CD site-specific mucosal inflammation.

## Introduction

Crohn's disease (CD) is a form of chronic auto-inflammatory bowel disease (IBD) characterized by patchy involvement of the intestinal tract. Although CD can involve any part of the intestine, ileo-colonic involvement is most common [1], [2]. Approximately 20–30 percent of all CD patients are children. Childhood presentation and subsequent treatment of CD may dramatically impact the patient's growth, development and overall quality of life [1], [3]. CD is pathogenetically based on prolonged remitting/relapsing inflammation of immune origin, which generates damage at local mucosal sites and includes systemic involvement. Immunological, genetic and environmental factors could stochastically overlap in triggering and perpetuating the inflammatory processes [4]. This study addresses the hypothesis that local inflammation is the outcome of inappropriate immune responses to common environmental stimuli, and that such responses contribute to disease activity independently of the events that have triggered the disease [2], [4], [5]. Such antigens should be available within both the microbial flora and the target tissue, over-expressed at the site of inflammation [6], [7] and strongly antigenic [8], [9], [10]. A growing body of work [8], [11]–[14], including our own published findings [15]–[17], implicate that heat shock proteins (HSP) are among the antigens capable of sustaining such immune/autoimmune inflammation.

We have demonstrated in various autoimmune diseases that HSP-derived epitopes are capable of inducing and modulating specific T-cell responses and that such modulation correlates with disease activity [15], [18], [19]. CD constitutes an ideal disease model to test this hypothesis, as it often presents with patchy intestinal involvement [1], [3], enabling us to compare inflamed and non-inflamed areas within the same individual, at the same time point. In the present study, we tested three pediatric populations - CD, Ulcerative Colitis (UC) and normal healthy patient biopsies.

These patient groups were tested for immune responses to a pool of HSP-derived peptides designed to be Pan HLA-DR binders (in order to overcome variability in presentation due to MHC polymorphisms). These peptides were engineered to be T-cell epitopes to focus on T-cell-mediated responses. Mucosal biopsies from inflamed and non-inflamed areas (as well as control patients without CD) were obtained and probed for production of cytokines involved in modulation of the immune response. Immunological data were correlated with clinical and histological data pertaining to disease activity.

## Results

### HSP60/65 peptide selection

The selection of the HSP60/65-derived peptide was performed using a mathematical algorithm as described in Sette et al. [20]. A list of peptides predicted to be good Pan-DR binders was generated. Affinity to 15 different HLA types was tested in binding assays for four human/bacterial homologous peptide pairs, including the ones described here [19]. Preliminary studies showed that the pairs P1–P2 and P7–P8 (see Table 1) were the most antigenic of the pool for pediatric CD patients (not shown).

**Table 1:** HPS60/65-derived peptides included in the study.

Name	Species	Accession N°	aa position	aa sequence
**P1**	*mycobacterium tuberculosis*	CAA17397.1	254–268	GEA**LSTLVVNKI**RGT
**P2**	*Homo sapiens*	AAH02676.1	280–294	GEA**LSTLVLNRL**KVG
**P7**	*mycobacterium tuberculosis*	CAA17397.1	507–521	IAGL**F*LTTEAVVA**D***K
**P8**	*Homo sapiens*	AAH02676.1	535–549	VASL**LTTAEVVVT**EI

Pan-DR binding motives are highlighted in bold underlined or bold italics when more than one Pan-DR-binding site is present. P1-P8 refers to the name give to the chosen peptides, aa: amino-acid.

### Proinflammatory reactivity to HSP-derived peptides is found in inflamed but not in normal mucosa in CD patients, UC patients or healthy patients

To assess the presence of specific immune reactivity against HSP-derived peptides, we analyzed biopsies of colonic mucosa from pediatric patients with CD, UC or no inflammatory disease by measuring cytokine mRNA levels using Quantitative Real-time polymerase chain reaction (QRT-PCR). In preliminary experiments, we were not able to extract sufficient T cells from the biopsy to perform functional assays, given the small size and the difficulty of obtaining multiple biopsies from the same pediatric patients.

Hence, we relied on HSP-derived peptides which were designed to be exclusively T-cell epitopes. We used such peptides as antigens in cultures employing the whole biopsy with the assumption, backed by published experience [16], [19], [21], that immune responses to these T-cell epitopes would be T-cell driven. Operators were blinded to the origin of the samples. We investigated whether the altered immune response to HSP60 was confined to chronically inflamed intestinal areas. Media alone served as the culture control. As mentioned above, CD is an ideal model of disease where both types of tissue, i.e. inflamed and normal, can be found in the same subject. Biopsies were classified macroscopically at the time of collection, and later analyzed microscopically by the pathologist for disease classification and scoring.

**Figure 1:**
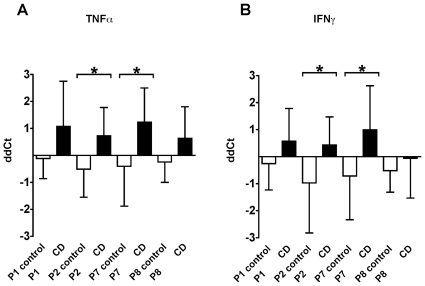
Mucosal proinflammatory response to HSP60/65-derived peptides in abnormal mucosa in the CD patients. CD tissue (black bars) and control tissue (open bars) were stimulated ex-vivo with P1, P2, P7 and P8. After 36 hours, the RNA from cultured biopsies was extracted to determine cytokine gene expression by QRT-PCR and values were expressed as ddCT. Figure 1A represents the ddCT for TNF〈 and Figure 1B represents the ddCT for IFN©. *p<0.05. Bars represent mean and SD of repeated tests.

CD patients showed a well-characterized individual repertoire of proinflammatory immune reactivity against specific HSP60/65-derived peptides as illustrated in Figure 1. This proinflammatory activity is only concentrated in sections of the intestine where the inflammatory infiltrate is present (black bars) as assessed by histological analysis. Reactivity was not detectable in the section of the intestine where the inflammation is minimal or absent (white bars). The inflamed tissue showed a specific immune reactivity directed mainly toward P2 and P7 peptides when compared to the non-inflamed tissue of the same patient group. Indeed, results showed that ex-vivo cultures of inflamed colonic biopsies re-stimulated with these peptides were able to consistently induce a proinflammatory cytokine profile dominated by increased mRNA levels of TNF〈 and IFN© as measured by QRT-PCR (Figure 1). The response in the CD patients to P2 and P7 for TNF〈 production showed statistical significance in abnormal biopsies (P 0.0122 and 0.0129 respectively) (Figure 1A). As shown in Figure 1B, we found that the human P2 and P7 peptides were also able to significantly induce IFN© in biopsy samples of CD patients (P 0.0267 and 0.0212 respectively).

### Proinflammatory reactivity to P2 and P7 peptides is characteristically disease- and site-specific

Peripheral blood mononuclear cells (PBMC) from the CD patients tested with the HSP-derived peptides had the same responses as normal controls (not shown). A comparison of patients with ulcerative colitis (UC) disease or healthy controls showed no differences in the induction of proinflammatory cytokines in response to stimulation with P2 and P7. However, the UC patient group showed reactivity to a different group of HSP-derived peptides (not shown). Disease-specific recognition patterns for HSP-derived T-cell epitopes is a phenomenon we have described in several autoimmune diseases and was confirmed here [12], [15]–[17].

### Correlation of immunological response between homolog peptide pairs

**Figure 2:**
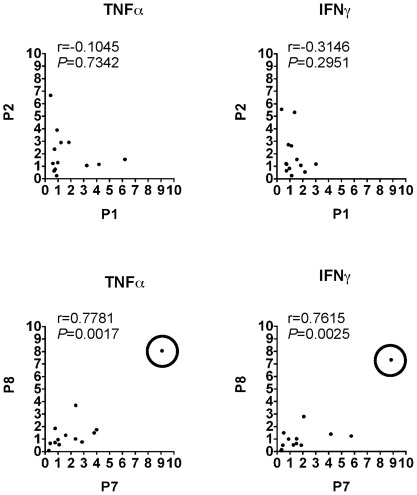
Correlation of proinflammatory cytokine response between HSP60/65-derived homolog pairs. Logarithmic values of QRT-PCR results of each HSP60-derived pair were correlated using Pearson correlation, n = 13 and a 95% confidence interval. Circles enclosing data points are shown to indicate the patient excluded, in secondary analysis, to demonstrate that correlations are not due to outliers.

As mentioned above, the selected peptide pairs included in the study originated from conserved regions of microbial HSP65 and the corresponding human homolog as Pan-DR binding peptides (Table 1). We performed a series of statistical correlations between the proinflammatory responses induced by the homolog peptide pairs to assess the concomitant presence of reactivity to the selected peptide pairs, P1–P2 and P7–P8. We found a strong association between P7 and P8 for the production of TNF〈 and IFN©. Pearson r-values were 0.7781 (P 0.0017) and 0.7615 (P 0.0025), respectively. This correlation was maintained even when the calculated outliers (enclosed in an open circle in Figure 2) were taken out of the analysis. The calculated r-values without the outliers were 0.6798 (P 0.015) and 0.6582 (P 0.0200) for TNF〈 and IFN©, respectively. No correlation was identified between the pair P1–P2 under the same statistical criteria (Figure 2).

### Immune response to HSP60/65-derived peptide in inflamed colonic mucosa are skewed toward an inflammatory rather than a regulatory phenotype

The expression pattern of IL4, IL10 and TGF® mRNA levels was analyzed following peptide-specific stimulation. None of the peptides included in the study were able to induce statistically significant differences in the expression level of IL4, IL10 and TGF® when cytokines were analyzed individually. IL10 induction between CD patients versus controls showed no statistically significant differences between the groups, with P-values of 0.6767; 0.3891; 0.2718 and 0.9829 for P1, P2, P7 and P8, respectively. Similarly, no statistical difference was found between control and CD for the TGF® and IL4 induction (not shown).

The ratio between IL10 and TNF〈 resulting from HSP60-derived peptides stimulation was determined to quantitatively assess the balance between regulatory and inflammatory functions in the intestinal mucosa of normal individuals versus CD patients. Interestingly, the IL10/TNF〈 ratio between normal control and CD groups were statistically significantly different for HSP60-derived peptide stimulation with P1, P7 and P8. The P-values obtained by comparison of the study populations were 0.0137, 0.0162 and 0.0406 for P1, P7 and P8, respectively. Conversely, no statistically significant difference was found for P2, perhaps due to the size of the standard deviation in the control group (Table 2).

**Table 2:** IL10 to TNF〈 ratio deduced from epitope-specific cytokine production of biopsies of control and CD patients.

	IL10/TNFα ratio		
Peptide	Control (n = 11)	CD (n = 12)	P-value
**P1**	1.29 (1.26)	0.95 (0.95)	*0.0137
**P2**	1.12 (17.68)	0.99 (0.76)	0.2345
**P7**	1.39 (2.07)	0.97 (1.26)	*0.0162
**P8**	1.19 (2.19)	0.93 (0.50)	*0.0406

Ratios are expressed as median (range) and P-values calculated by unpaired t-test analysis between groups. Positive significant correlation is shown with *.

### Immunological proinflammatory responses to HSP-peptides correlate with disease activity

The Pediatric Crohn's Disease Activity Index (PCDAI) [22] was used as an objective clinical parameter to investigate a possible pathogenic relevance for the proinflammatory responses to HSP60/65 epitopes and determine whether there was a statistical correlation between the induction of a proinflammatory response by specific HSP60/65-derived peptides and disease severity.

**Figure 3:**
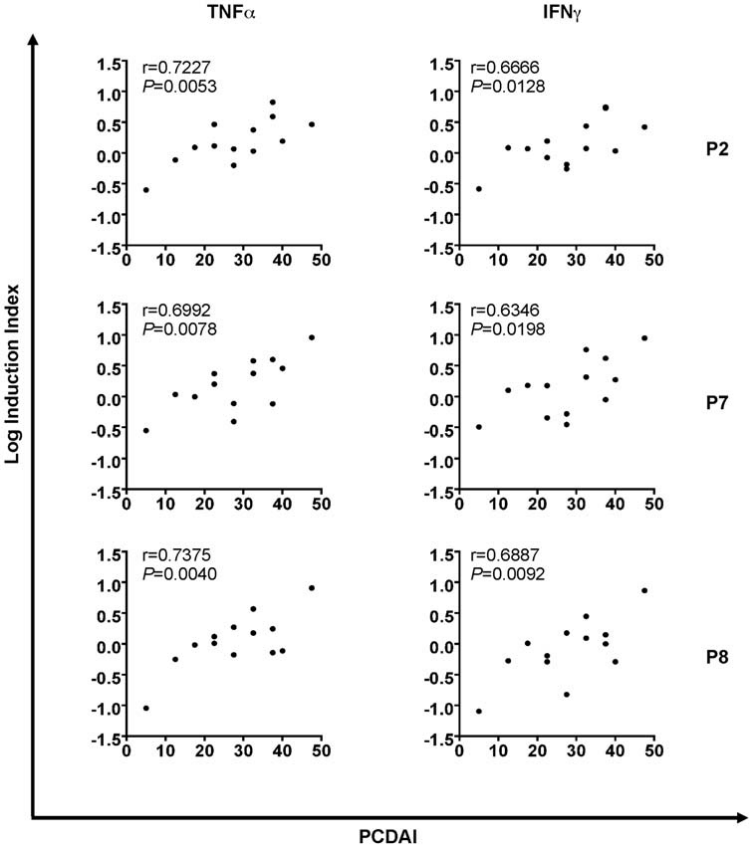
Correlation between immunological data and disease activity. Logarithmic values of QRT-PCR results were correlated with PCDAI by using Pearson correlation. Left and right panels correspond to TNF〈 versus PCDAI and IFN© versus PCDAI correlations, respectively.

Indeed, data analysis showed a statistical correlation between PCDAI in CD patients with P2 and P7 peptide cytokine production that proved to be relevant in the immunological studies, i.e., the HSP60/65-derived peptide that triggered a prevalent proinflammatory cytokine induction. As shown in Figure 3, P2 showed a positive Pearson r-value of 0.6666 and 0.7227 for IFN© and TNF〈, respectively. P-values for these correlations were also statistically significant, 0.0128 and 0.0053, respectively. Similarly, peptide P7 revealed r- and P-values of 0.6992 and 0.0078, respectively, for TNF〈 while r- and P-values of 0.6346 and 0.0198 for IFN© (Figure 3). No correlation was found between proinflammatory response and disease duration. The QRT-PCR results for TNF〈 and IFN© for the P8 peptide presented a high statistical correlation with PCDAI with Pearson r-value of 0.7375 (P 0.0040) and 0.6887 (P 0.0092), respectively. P1 showed no correlation to disease activity.

### Immunological proinflammatory responses to HSP-peptides correlate with histologic data

In further support of the relevance of the correlations between the immunological findings and the clinical picture, we performed statistical correlations between the proinflammatory response directed against the relevant HSP60-derived peptides and histological scores of the biopsies.

**Figure 4:**
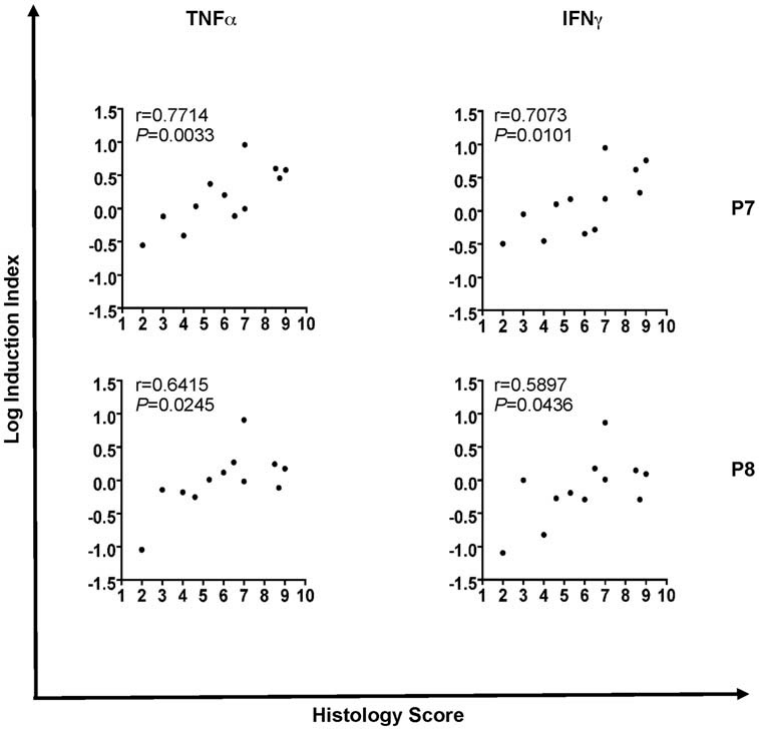
Correlation between immunological data and histology score. Logarithmic values of Induction Index were correlated with histology score by using Pearson correlation. Left and right panels correspond to TNF〈 versus histology score and IFN© versus histology correlations, respectively.

A positive statistical correlation was found with P7 and P8 peptides when proinflammatory cytokine QRT-PCR values were associated with histological score in CD. We used a Pearson statistical correlation with all peptides and the individual histological scores provided by the pathologist. Positive correlations of 0.7073 (P 0.0101) and 0.7714 (P 0.0033) for P7 were found when histological score was compared to IFN© and TNF〈 logarithmic Induction Index, respectively. Similarly, positive correlations were found for P8, with r-values of 0.5897 (P 0.0436) and 0.6415 (P 0.0245) for IFN© and TNF〈 induction, respectively (Figure 4).

### IFNγ and TNFα are CD4 positive producing T cells in the biopsies of inflamed CD mucosa

**Figure 5:**
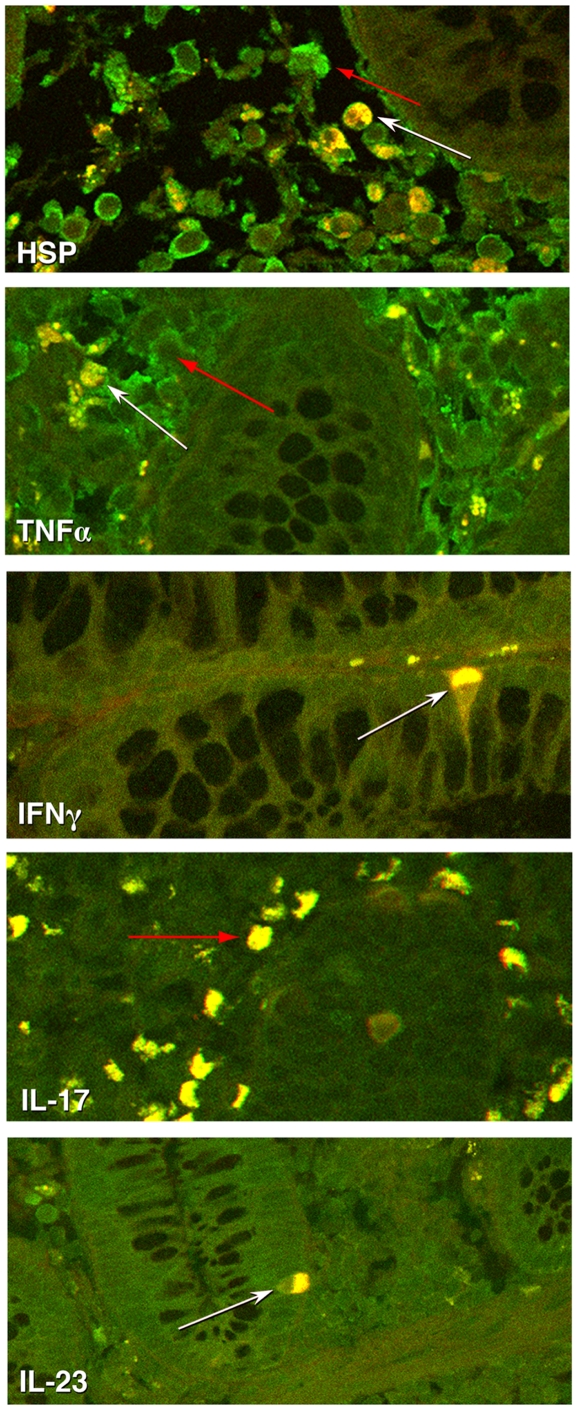
CD4 T cells produce TNFα, IFNγ, IL23 and HSP 60. Immunohistochemical analysis of inflamed CD sections shows the colocalization of TNFα, IFNγ, IL23 and HSP60 with CD4. CD4 FITC (shown in green) and TNF〈, IFNγ, IL23, or HSP60 (shown in red) can be detected in inflamed sections. Additionally, IL-17 (shown in green) producing cells can be seen in inflamed sections. White arrows depict a representative double labeled cell and red arrows depict a representative single labeled cell.

To validate the QRT-PCR data and to assess the contribution of T cells to the local inflammatory process, immunohistochemistry was performed (Figure 5). Analysis of inflamed and normal biopsies indicated that IFNγ and TNFα producing cells were CD4 positive T cells with an increased amount of colocalization found in inflamed tissue from patients with CD. In addition, inflamed biopsies showed upregulation of IL23 and an abundance of HSP60 expression in CD4 positive T cells. Only inflamed tissue expressed IL17. IL-10 and FoxP3 expression was not detected in the biopsies (data not shown).

## Discussion

Despite of recent advances in the clinical understanding of CD, important questions concerning the immunopathology of this disease remain unanswered, particularly regarding the cause of persistent gut inflammation. A commonly held view is that the chronic inflammation is primarily the consequence of a disregulated adaptive immune system leading to an immunological imbalance with a resulting excess of proinflammatory cytokines [5], [23], [24].

Our working hypothesis aims to address the question of whether the inflammation of colonic mucosa in pediatric CD is the result of an inappropriate and self-perpetuating immune response to endoluminal antigens, perhaps in association with overlapping failures of the immunoregulatory mechanisms of inflammation [25]–[27]. To perpetuate inflammation, such immune responses should ideally be detectable at the site of mucosal inflammation and directed toward antigens readily available and possibly over-expressed at such sites [6], [7], [9]. Perpetuation of proinflammatory responses could also be, at least in part, determined by cross recognition of sequence motifs shared between self and exogenous proteins [28]–[30]. This would be particularly important in the context of a disease, such as CD, where there is an abundance of exogenous antigens in direct contact with the inflamed tissues [25], [31], [32]. The need to assess specificity of antigen recognition led us to focus on adaptive immune responses of T cells. Consistent with these postulates and our own previous work [16], [17], we evaluated immune responses to HSP and correlated such responses with clinical and histological characteristics of the disease. The scarcity of the samples due to the age of the subjects and the consequent impossibility of obtaining enough purified T cells posed a technical hurdle. Hence, we stimulated the whole biopsy with peptides, which were designed and validated to be T-cell-specific epitopes, to allow for the study of T-cell-specific responses.

The focus of this study included pairs of bacterial/human homologue peptides derived from HSP60/65, which were designed to be Pan HLA-DR binders to overcome inconsistencies in immune responses determined by HLA polymorphism. This method has proved to be very successful in identifying T-cell-specific epitopes relevant to the immunopathogenesis of various diseases [16], [19]. The use of both inflamed and normal mucosa from the same patients allowed efficient measurement of T-cell-specific immune responses directly related to the inflammatory process. This approach was effective in identifying molecular signatures of immunological responses to HSP in CD and in correlating them with disease activity and histological aspects of inflammation. In fact, we found that two of the peptides tested, namely P2 (human-derived) and P7 (bacterial-derived) induced a significant increase in proinflammatory immune responses in CD patients when compared to controls. This reactivity was not found in samples from non-inflamed mucosa from the same CD patients, UC patients and healthy controls. Furthermore, the same peptides were unable to significantly induce TNF〈 and IFN© in PBMC from the same patients, which supports the idea that recognition is restricted to the inflammation site. Hence, immune responses to HSP60 epitopes are disease- and inflammation-specific. Proinflammatory immune responses to P2, P7 and P8 significantly correlate with clinical and histological scores of disease activity. These findings underscore the potential that abnormal immune responses to HSP may affect the clinical relevance of the disease, thus suggesting a direct pathogenic relationship. At least in this series, proinflammatory immune responses did not correlate to disease duration in the CD patients studied.

HSP responses in human autoimmunity are exceptionally complex [10], [33] and are likely the result of a dynamic balance between self and non-self recognition in the context of apparently contradictory proinflammatory and tolerogenic signals [14], [34]. This network of immune responses is the consequence of the abundance of peptides with significantly conserved sequence homology at the site of inflammation. This homology can often lead to cross reactivity between self and non-self proteins [14], [18], [28]–[30]. In the case of CD, we found a significantly positive correlation between human and microbial peptides for their ability to induce proinflammatory responses, supporting the concept of immune cross reactivity as one of the mechanisms fueling inflammation. As we recently suggested [17], the complexity of immune responses to HSP-derived peptides underscores the role in which immunity to HSP plays in the pathogenesis of autoimmune inflammation in various diseases, irrespective of the disease triggers [12], [19], [35], [36]. Indeed, the immunological responses of proinflammatory epitopes in CD significantly correlate with clinical and histological signs of active inflammation. The human-derived peptide P2 responses can be directly classified as autoimmune. The combination immune responses found with the P7/P8 pair may be the result of overlapping mechanisms of cross reactivity that perpetuate inflammation.

We could not find significant differences for IL10 induction between controls and CD patients after peptide stimulation in ex-vivo cultures of colonic biopsies; suggesting that at least at this level, the regulatory cytokine profile is similar between groups. However, the ratio of IL10/TNF〈, which is commonly considered an indicator of overall balance between inflammatory and regulatory functions [19], was statistically different between controls and CD for most of the tested peptides. Such a finding suggests that a dysfunctional balance is present in the complex regulatory mechanisms involved in the chronic inflammation seen in CD. Indeed, it has been shown that clinical response to infliximab in UC patients was correlated to reduction in TNFα mRNA and additional reduced expression was seen for IFNγ, but not that of IL10 and IL4 mRNA [37].

Immunohistochemistry confirmed an increase in the inflamed versus noninflamed biopsies for the pro-inflammatory cytokines; TNF〈, IFN©, IL17 and IL23. This upregulation was contributed by CD4 positive cells, which were abundant in inflamed biopsies, indicating specific CD4 T cell involvement in chronic inflammation of IBD.

In addition, inflamed biopsies showed an abundance of HSP60 expression, including in CD4 positive cells, confirming visually a direct link between immunologically relevant cells and expression of HSP in the presence of chronic inflammation. These data confirm a plethora of reports supporting the upregulation and immunodominant role for HSP in inflammation. This notion does not necessarily collide, in our opinion with a recent report from Hu et al. These investigators reported a decrease in HSP27 and HSP70 expression in actively inflamed mucosa [38]. HSP27 and HSP70 have different regulatory mechanisms than HSP60 (or dnaJ); and in that specific study, their expression was not measured in immune cells [38]–[40].

In summary, our data propose an epitope-specific contributor of autoimmune inflammation in CD, define the dominant epitopes and consequently may support a future translational approach that, based on the possibility to design an epitope-specific immunonotherapeutic intervention in pediatric CD patients, could be an alternative treatment able to restore normal regulatory immune functions [41] as seen in other autoimmune disease models [42].

## Materials and Methods

### Patient population

Patients were recruited from the pediatric gastroenterology outpatient and inpatient services at Children's Hospital, San Diego. Healthy patients and IBD patients undergoing colonoscopy for varying routine indications were asked to participate in this study. The Human Research Protection Programs of University of California San Diego and Children's Hospital of San Diego approved consent forms were completed for each enrolled subject. IBD Study Group: Inclusion criteria were children between 3–17 years of age, with IBD undergoing colonoscopy as part of their initial or follow-up evaluation. Exclusion criteria were the evidence of concurrent gastrointestinal infection, allergic colitis or any other chronic disease involving the intestinal tract. Healthy Control Group: Inclusion criteria were children undergoing colonoscopy for specific gastrointestinal symptoms, but who were found to have normal endoscopy and histology, anal fissures or single juvenile polyp.

### Study Subjects

No significant differences in age, gender or ethnicity were detected in the patient population used. The study population was composed as follows: 11 subjects in the healthy patient control, 13 in the CD and 13 in the UC group.

### Clinical Procedure

All procedures were performed under general anesthesia by a pediatric gastroenterologist. Biopsies were taken through the Olympus PCF100 colonoscope (Melville, NY) using disposable multiple sample biopsy forceps (Boston Scientific, Watertown, MA).

### Disease activity index and Histology

In patients with CD, clinical disease activity was evaluated using the Pediatric Crohn's Disease Activity Index (PCDAI) [22] at enrollment. For histological examination, colon biopsies were fixed using standard processing on formalin-fixed paraffin embedded tissue. Biopsy sections were stained with Hematoxylin 2 and Eosin Y (Richard-Allan Scientific Kalamazoo, MI), according to manufacturer's instructions. Analyzed tissues were scored for the degree of inflammation as follows: Acute inflammation (0 normal, 1 mild, 2 moderate and 3 severe); Chronic inflammation (0 normal, 1 mild, 2 moderate and 3 severe); Ulceration (0 present and 1 absent); and Crypt abscesses (0 present and 1 absent). The maximum histology score was 8. The pathologist was blinded to patient's clinical history.

### Peptide selection

All HSP60/65-derived peptides were designed using a computing algorithm developed by Dr. Sette (LIAI, La Jolla, CA) [20] and validated by using HLA-DR in binding assays [19]. All peptides used in this study were HSP60/65-derived peptides (Synthetic Biomolecules Inc., San Diego, CA) of 15 amino acid length originated from the conserved regions of human and Mycobacterium tuberculosis HSP60/65. Due to limitations of biopsy sample size, only two sets of peptides were chosen for this study and are shown in Table 1. Additionally, the P1/P2 and P7/P8 homolog pairs were chosen due to their optimum Pan DR scores in the microbial and human epitopes, respectively [19].

### Biopsy stimulation

The operator, blinded to clinical and histological reports, performed the peptide stimulation of biopsy sample in addition to immunological analysis. Whenever possible, two sets of biopsies per patient were obtained from endoscopically normal and abnormal colonic mucosa at gross appearance. Biopsies were washed once in fresh media; cultured directly in U-bottom 96-well plate in a final volume of 200 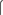
l for 36 hours in complete media (RPMI 1640, 10% heat-inactivated serum, Penicillin, streptomycin, and L-Glutamine) in a 5% CO_2_ incubator at 37°C. Incubation with HSP60/65-derived peptides was done at 10 µg/ml or with phytohemagglutinin (PHA) at 2.5 µg/ml. Samples were disrupted by adding Lysis buffer and frozen at −80°C up to 1 month before RNA extraction.

### Quantitative Real Time PCR assessment of cytokine expression

Total RNA was extracted from biopsies incubated with either media or HSP60/65-derived peptide. The total RNA was reverse transcribed using Improm II Reverse Transcriptase strand synthesis system (Promega, Madison, WI,) in a final volume of 20 µL. The relative amount of TNF〈, IFN©, IL10, TGF® and IL4 mRNA in each sample was normalized to glyceraldehyde 3-phosphate dehydrogenase (GAPDH). Each measurement was carried out in duplicate at the linear amplification range of the QRT-PCR. Results were expressed as the ddCT of peptide stimulation and media control. The forward and reverse primers and probes (IDT, Coralville, IA) used for the PCR were as follows: GAPDH, GeneBank Accession number (Acc. N°) M33197: (212–230), (259–280) and JOE (231–253) BHQ-1; IFN©, Acc. N° NM_000619: (492–511), (546–567), JOE (518–542) BHQ-1; IL4 Acc. N° NM_000589.2: (645–663), (692–710), FAM 5′ (665–689) BHQ-1; IL10, Acc. N° NM_000572: (137–157), (186–206), FAM 5′ (160–185) BHQ-1; TGF®1, Acc. N° NM_000660.1: (1728–1747), (1772–1789), TET (1749–1768) BHQ-1 and TNF〈, Acc. N° NM_000594: (775–794), (826–843), FAM (796–820) BHQ-1, respectively. Immunological analyses presented in the study were performed blinded to clinical and histological reports.

### Immunohistochemistry of CD biopsies

Formalin fixed and paraffin-embedded biopsies were used for immunohistochemical analysis of TNF〈, IFN©, IL10, HSP60, IL23 and CD4 (AbCam). 10 µM sections from both inflamed and normal tissue biopsies were used from seven patients for analysis. Briefly, sections were deparaffinized in xylene. The slides were rinsed and antigen retrieval was performed using Dako target retrieval solution for 30 minutes at 96°C. Sections were blocked for two hours and stained with primary antibody for an additional two hours. Slides were then incubated in fluorescent secondary antibody from Invitrogen. Following three washes, slides were coverslipped using Vectashield from Vector Laboratories and viewed on a Zeiss confocal microscope.

### Data Analysis

Statistical analysis was performed using GraphPad Prism (GraphPad Software, San Diego CA). When necessary, a log transformation was performed to obtain a normal distribution. Parametric t-test was used for between-groups comparisons. In all cases, log transformation was used at a confidence interval of 95%. A P value of <0.05 was considered significant. Two-tailed evaluation was always applied for the statistical tests. Pearson correlation coefficients were calculated to assess relationships between Induction Index and PCDAI or Histologic score or in between peptides.
